# MS-ANet: deep learning for automated multi-label thoracic disease detection and classification

**DOI:** 10.7717/peerj-cs.541

**Published:** 2021-05-17

**Authors:** Jing Xu, Hui Li, Xiu Li

**Affiliations:** 1 Shenzhen International Graduate School, Tsinghua University, Shenzhen, Guangdong, China; 2 Institute of Computing Technology, Chinese Academy of Sciences, Beijing, China

**Keywords:** Multi-label, Chest X-Ray images, Multi-Scale Attention Networks, Image Classification

## Abstract

The chest X-ray is one of the most common radiological examination types for the diagnosis of chest diseases. Nowadays, the automatic classification technology of radiological images has been widely used in clinical diagnosis and treatment plans. However, each disease has its own different response characteristic receptive field region, which is the main challenge for chest disease classification tasks. Besides, the imbalance of sample data categories further increases the difficulty of tasks. To solve these problems, we propose a new multi-label chest disease image classification scheme based on a multi-scale attention network. In this scheme, multi-scale information is iteratively fused to focus on regions with a high probability of disease, to effectively mine more meaningful information from data. A novel loss function is also designed to improve the rationality of visual perception and multi-label image classification, which forces the consistency of attention regions before and after image transformation. A comprehensive experiment was carried out on the Chest X-Ray14 and CheXpert datasets, separately containing over 100,000 frontal-view and 200,000 front and side view X-ray images with 14 diseases. The AUROC is 0.850 and 0.815 respectively on the two data sets, which achieve the state-of-the-art results, verified the effectiveness of this method in chest X-ray image classification. This study has important practical significance for using AI algorithms to assist radiologists in improving work efficiency and diagnostic accuracy.

## Introduction

Common chest diseases such as pneumonia and lung cancer are threatening human life and health. Many chest lesions, such as nodules and emphysema, are early manifestations of lung cancer. Lung cancer is one of the main causes of human death due to cancer, which causes about 4 million deaths each year (Ruuskanen et al., 2011). Because lung cancer may be infected by simple chest diseases, there is an urgent need for lung cancer screening, early detection and personalized treatment. Chest X-ray examination is the most commonly used radiological examination in screening and diagnosing chest lesions, and computer-aided X-ray analysis has been widely used. However, it is difficult to obtain multi-label classification data of radiological images and each disease has its own different response characteristic receptive field region, which is the main challenge for chest disease classification tasks. Also, the imbalance of sample data categories further increases the difficulty of tasks.

To solve the above problems, this paper takes the medical AI as the background, applies the advanced deep learning technology to the medical scene, and explores an efficient and accurate multi-label classification algorithm for deep medical images to help doctors quickly identify lesions, which can greatly improve the efficiency and accuracy of clinical diagnosis.

## Related work

Most chest X-ray datasets are composed of multi-classes whose positive/negative sample ratio is unbalanced. Also, dominant samples are mostly simple and easy classes, and different diseases focus on different characteristic regions. Therefore, the automatic classification of chest diseases is still challenging.

To solve these problems, domestic and foreign scholars have proposed many advanced computer vision algorithms. Dai et al. (2020) proposed a multi-scale channel attention module, which adds the local channel context to the global channel statistics, and solves the problems when fusing different scale features. Chen et al. (2020) proposed a new CheXGCN structure based on Graph Convolution Network (GCN), where the co-occurrence and interdependence of labels were employed for multi-label CXR image classification, improving the recognition accuracy. Lin et al. (2017) proposed Focal Loss, which modifies cross-entropy loss by adopting a modulation term to focus on learning on complex samples and mitigate many easy negative effects.

In the field of multi-label chest disease classification research, Ho & Gwak (2019) proposed a novel framework to integrate multiple features from both shallow and deep features, representative and discriminant features are extracted from open ChestX-ray14 data sets to distinguish 14 pathological types. Liu et al. (2020) proposed a new semi-supervised framework for medical image classification based on relation-driven, which utilizes unlabeled data by stimulating the prediction consistency of a given input under disturbance and generates high-quality consistency targets for unlabeled data by using a self-assembly model. Ma, Wang & Hoi (2019) proposed the cross-attention network (CAN) framework, which mined effective representations from the data through mutual attention and updating the model in a more collaborative manner. Despite the great success of many medical image application methods, there is no good solution to the problem that the corresponding parts of the visual presentation of the multi-label classification model before and after image transformation are inconsistent for different diseases.

Therefore, we propose a multi-scale information fusion network based on attention from two different perspectives to solve the problem of multi-label chest X-ray image classification. Helping doctors identify lesions quickly can significantly enhance the clinical diagnosis efficiency and accuracy.

1. From the perspective of image processing, a multi-scale attention network framework is designed, which is a flexible learning framework that can mine more meaningful information in an end-to-end manner. Because different diseases need to pay attention to different places and their receptive fields are different, only the full use of multi-scale information can better learn discriminative features adaptively to improve the classification accuracy.
2. From the perspective of visual perception and data distribution, a new loss function is proposed, which consists of a perceptual loss and a multi-label balance loss. The former can help the model learn better visual consistency feature representation, and the latter can reduce the imbalance between negative and positive categories in each class, controlling simple class samples. Also, image localization labels of some diseases are used to verify our model, which proved that the model could better locate the high-risk pathogenic regions. The effectiveness of the proposed method is evaluated on CheXpert and Chest X-Ray14 public datasets.

The remainder of this paper is structured as follows. The proposed method multi-scale networks and loss function are described in “Related Work”. “Experiments” analyzes the experimental results on Chest X-Ray14 and CheXpert datasets. Lastly, “Conclusion” concludes this paper.

## Model

### Multi-Scale Networks

#### Feature extraction networks

The preprocessed input data is fed into two feature extraction networks, where the convolution layers are used as the feature extractor. The employed backbone models for these two feature-extraction sub-networks can be replaced by other models in the proposed flexible multi-scale fusion framework. Unlike the regular CNN, the proposed unified general scheme, namely MS-FIF (Multi-Scale Feature Iterative Fusion), iteratively fuses the feature results extracted from different layers.

**Figure 1:**
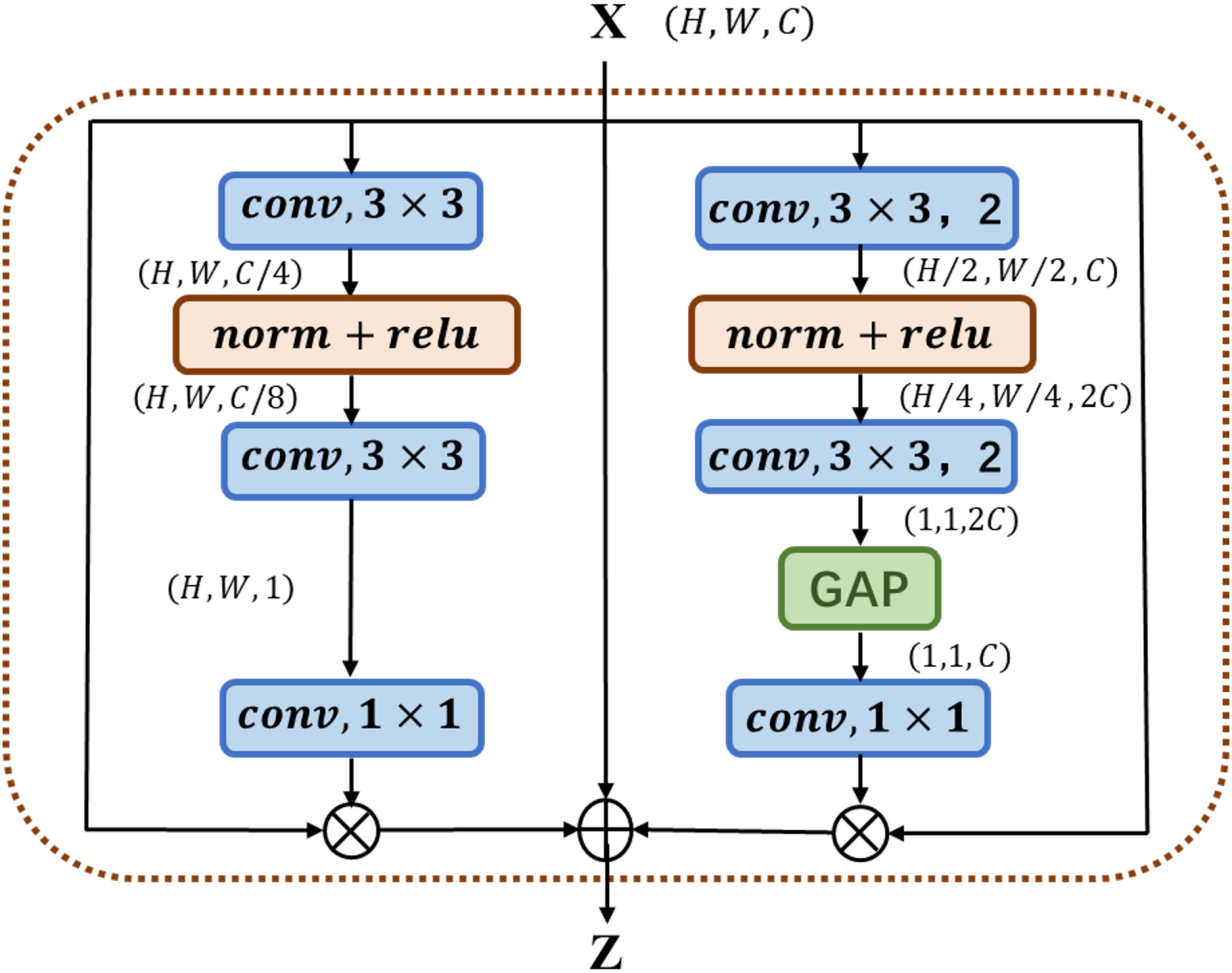
Illustration of the proposed MC-AB.

The multi-channel attention block MC-AB (Multi-Channel Attention Block), as shown in Fig. 1, has a simple structure and uses two branches with different scales to extract the channel attention weight. One branch extracts the spatial attention of local features, and the other uses Global Avg Pooling averaging to extract the channel attention of global features.

**Figure 2:**
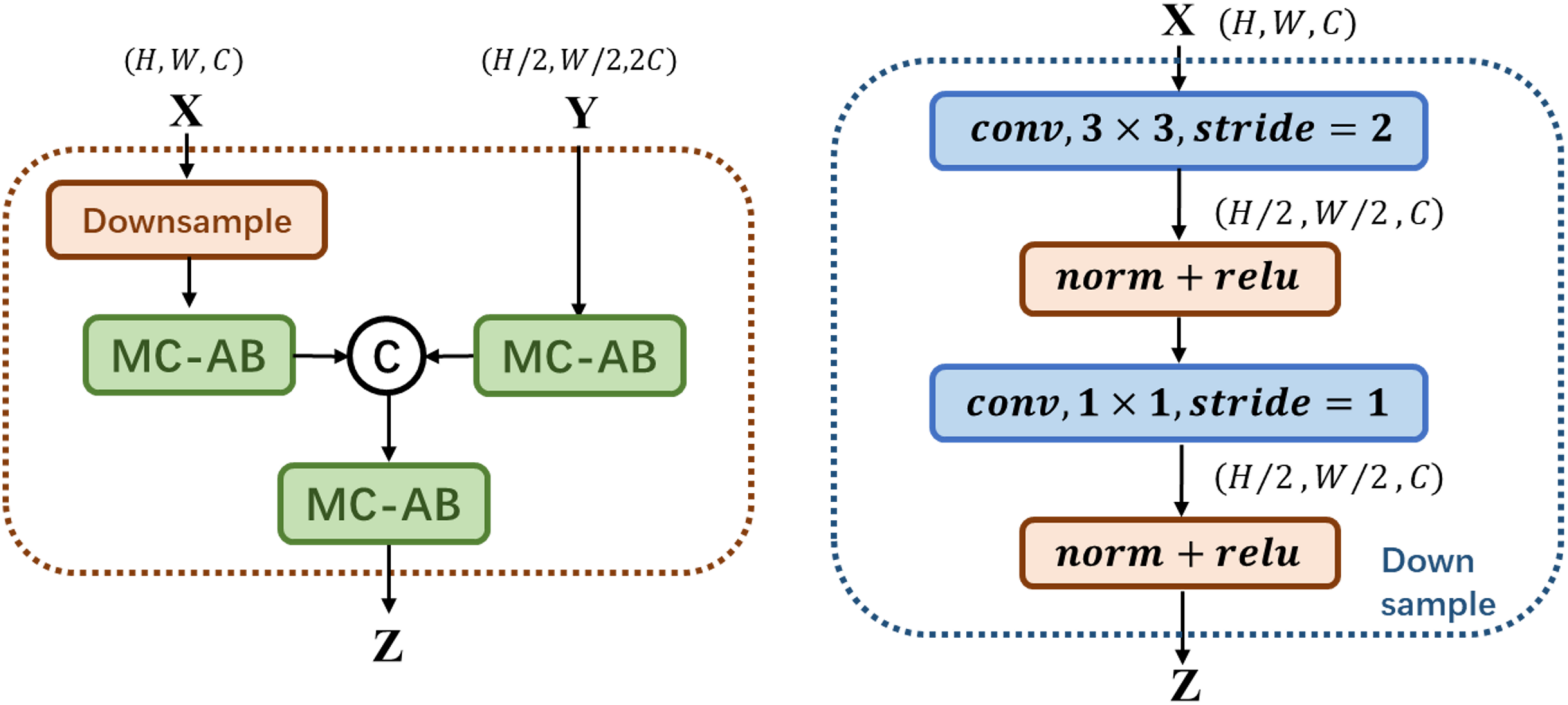
Illustration of the proposed MS-FIF.

As shown in Fig. 2 above, MS-FIF mainly focuses on the attention problem in the fusion of different scale features in different network structures. Given two feature graphs X,Y∈R, Y is a feature graph with a large receptive field range, where MC-AB is a multi-channel attention module, $Z\;\epsilon \;R^{C\times H\times W}$ is the fused output feature, C is CONCAT. The large-scale feature maps are down-sampled, so that the two feature maps are the same scale, connected after passing through the multi-channel attention module, and finally output after passing through a multi-channel attention module.

#### Multi-scale model

**Figure 3:**
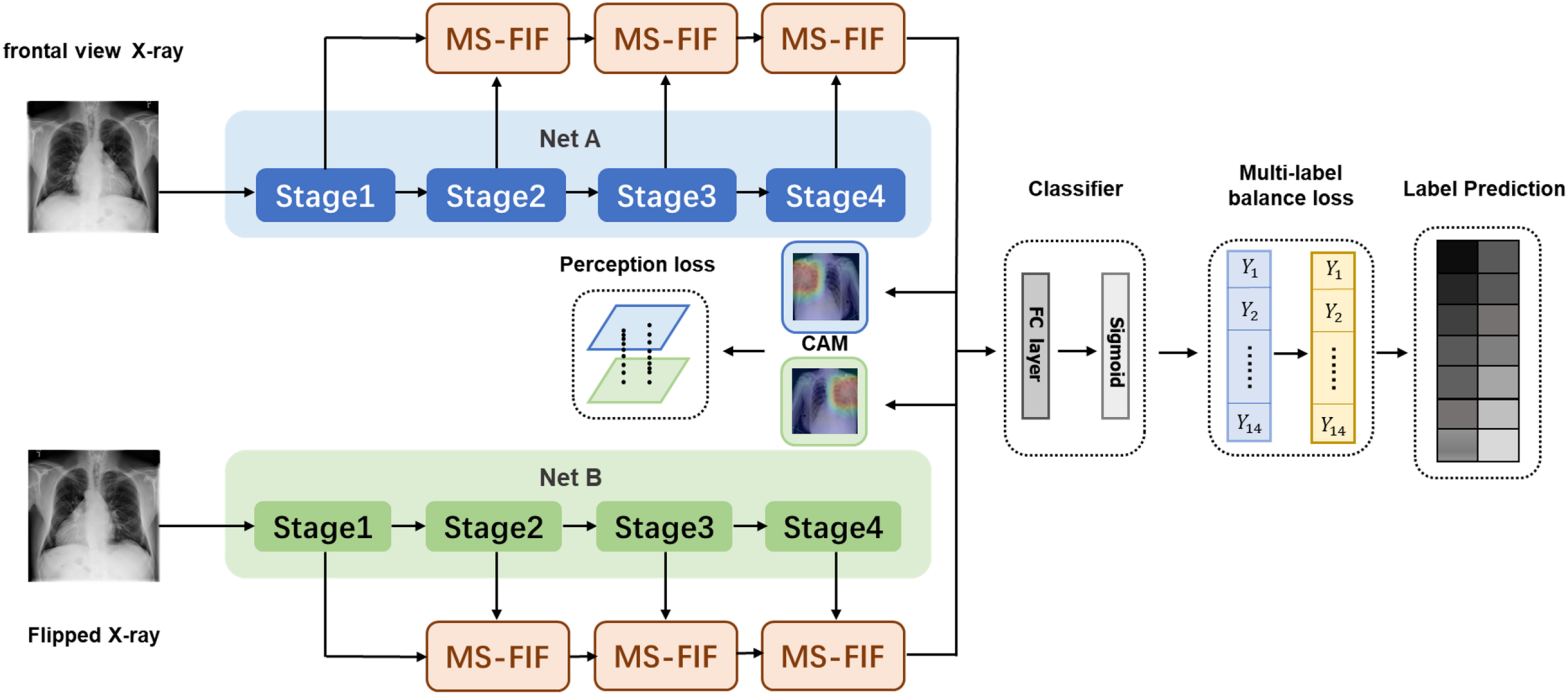
Multi-Scale Attention Networks. (1) MS-FIF is used to better iteratively fuse features; (2) using CAM to get the pathogenic attention map, prompting the network to focus only on areas with high pathogenic probability; (3) false frame represents the perceived loss and multi-label balance loss.

We propose a new multi-scale attentional network attention model, which combines the features of different levels or branches to obtain more meaningful features. In Fig. 3, The preprocessed original and rotated input images are fed into two networks with diverse initialization or structure, and fuse the features of four stages through MS-FIF iteration to better fuse semantic and scale inconsistent features. Then, the CAM (Class Activation Map) is used to get the disease attention map, which makes the network only focus on areas with high disease probability. Finally, output the results through the full connection layer.

Figure 3 Multi-Scale Attention Networks. (1) MS-FIF is used to better iteratively fuse features; (2) Using CAM to get the pathogenic attention map, prompting the network to focus only on areas with high pathogenic probability; (3) False frame represents the perceived loss and multi-label balance loss.

$F_{A}$ and $F_{B}$ represent the characteristic graphs of networks A and B. Through CAM, a feature map is formed by overlapping the feature set weighted by classifier full connection layer weight W, to better make classification decisions based on which feature maps are mainly from the feature set. The losses from two networks are propagated into each other during the backpropagation process, allowing collaborative updating of the networks.

#### Classifier

As depicted in Fig. 3, the feature maps obtained from the multi-scale attention networks are combined through the global average pooling and fed into a classifier. The proposed method consists of binary classifiers for each disease class. Each binary classifier consists of a fully-connected layer and a sigmoid activation function to compute the probability of being each chest disease.

### Loss function

#### Perceptual loss

A novel perception loss is defined to extract more significant features in the proposed model. As depicted in Fig. 3, all the features of the last layer of attention feature fusion are obtained in the two networks. Note that the value of each pixel in attention maps indicates different spatial information of the original image. The feature map is then averaged through CAM, and the response size of the feature map is mapped to the original map. The location of different diseases and the size of the lesion area varied greatly on X-ray images and the texture was diverse, and most of them were concentrated in the 'correct' area. A pathogenic attention map is roughly obtained by summing feature maps, computed as follows:

(1) $$S_{c}=\sum_{x,y}\sum_{k}w_{k}^{c}f_{k}(x,y)$$

where $S_{c}$ represents the CAM figure of the $C^{th}$ category, $w\in R^{c\times k}$ represents the weight of the full connection layer, k represents the number of channels, and $f\in R^{C\times H\times W}$ represents the characteristic figure.

For image x, $\hat{x}=T\left(x\right)$ is obtained after *T* transformation, and the corresponding CAM has $\hat{S}=T\left(S\right)$. We obtain two CAMs from the two networks before and after image transformation, S and $\overset{´}{S}$. The L2 loss of two CAM graphs is then computed. The attention loss constrains the cross-attention process smoother by forcing the two networks to find mutually pathogenic regions. The formula for perceived loss is as follows:

(2) $$L_{per}=\overset{c}{\underset{i}{\sum }}\frac{1}{cHW}{\left|\left|{\hat{S}}_{i}-\;{\overset{´}{S}}_{i}\right|\right|}_{2}$$

where c represents the number of categories for Label and $S_{i}$ represents the CAM diagram for the $i^{th}$ category.

#### Multi-label balance loss

The imbalance between data classes is not conducive for the network to master sufficient texture information; Many samples contain multiple disease information, which is difficult to train; The pathological information of different diseases is different, resulting in different degrees of difficulty in learning. Inspired by Lin et al. (2017), a multi-label balance loss is defined, which is an extension of the focus loss to support multi-classes by adding a balance factor $\alpha_{i}$, extracting more significant features from dominant samples while addressing the imbalance of samples. We aggregate the balance losses for each disease to represent the balance losses for multiple labels:

(3) $$L_{bal}=\overset{c}{\underset{i=0}{\sum }}\alpha_{i}{\left(1-\hat{y_{i}}\right)}^{p_{i}}log\left(\hat{y_{i}}\right)$$

where $\alpha_{i}$ denotes a factor that reduces imbalance between negative and positive samples for *i*^*th*^ class (*i* = 0…*c*), with a general value of $\frac{\left|x_{i}\right|}{\left|x\right|}$, where$\;x_{i}$ denotes the number of classes i. $\;\hat{y_{i}}\;\left(i=1,2,\cdots ,14\right)\;$represents the probability value (predictive value) of the network to determine the prevalence of the i disease. Parameter $\;P_{i}\;$ is a difficult-to-easy sample factor. Under certain $\;P_{i}\;$ settings, this may have better performance in mining “difficult” samples, thus making a greater contribution to model training, especially when “easy” samples occupy a large proportion of the data set.

#### Multi-scale perceptual loss

The multi-scale perceptual loss is defined as the combination of perceptual and multi-label balance losses as follows:

(4) $$L=\alpha L_{per}+L_{bal}$$

where α is a coefficient parameter that controls the trade-off between two losses. More weight on *L*_*per*_ induces a more accurate model, focusing on the region where the disease occurs. In contrast, more weight on the multi-label balance loss increases the loss weight of difficult-to-identify diseases and reduces the loss weight of easy-to-identify diseases, thereby enhancing the network's learning of difficult-to-identify samples.

## Experiments

### Results on Chest X-Ray14 dataset

#### Dataset and training

In this section, the proposed network is evaluated on the Chest X-Ray14 dataset (Wang et al., 2017), which comprises 112,120 frontal-view X-ray images of 30,805 unique patients with the text-mined fourteen disease image labels (where each image can have multi-labels), mined from the associated radiological reports using natural language processing. According to the official ratio, the dataset is divided into training and testing sets to ensure a fair comparison. In the experiment, we zoom the input image to 256 × 256, and randomly select the center point to cut the image to 224 × 224. Random rotation and random flip data enhancement methods are used in the training process. Among training data, 78,485 images and 8,039 images are used for training and validation, respectively. No overlap among the three patient-sets is ensured. The data batch size is 128, and the learning rate is initially set as 0.001. The dropout rate is set as 0.2 for the last fully-connected layer, and α is set to 0.0002. The $P_{i}$ in the multi-label balance loss is set to 1.5 according to the existing research. All experiments were evaluated based on the AUROC value.

#### Comparison analysis

Table 1 presents the quantitative comparison of the performance of different models. Multi-Scale Attention Network—MS-ANet_1_, MS-ANet_2_, and MS-ANet_3_ are constructed with different backbone networks. MS-ANet_1_ is the multi-scale attention network whose backbone networks are two densenet121 networks. MS-ANet_2_ represents the multi-scale attention network model with densenet121 and densenet169 backbone networks. MS-ANet_3_ represents the multi-scale attention network model with two densenet169 backbone networks. Each sub-network is differently initialized using distinct weights. Note that additional localization labels were used in the experiments of Li et al. (2018) in addition to the official segmentation data. For a fair comparison of the experiments, the results are not considered for the best results of each row (marked in bold).

**Table 1:** Results comparison between different methods on Chest X-Ray14 dataset.

Diseases	Wang et al. (2017)	Yao et al. (2017)	Rajpurkar et al. (2017)	Li et al. (2018)	Guendel et al. (2018)	Wang & Xia (2018)	Ma, Wang & Hoi (2019)	MS-ANet_1_	MS-ANet_2_	MS-ANet_3_
Split by Wang	Yes	Yes	Yes	Yes	Yes	Yes	Yes	Yes	Yes	Yes
Image resize	256*256	256*256	256*256	256*256	256*256	256*256	256*256	256*256	256*256	256*256
Atelectasis	0.773	0.733	0.759	0.800	0.767	0.743	0.777	0.823	**0.831**	0.829
Cardiomegaly	0.854	0.856	0.871	0.870	0.883	0.875	0.894	0.908	**0.910**	0.905
Effusion	0.861	0.806	0.821	0.870	0.828	0.811	0.829	0.882	**0.886**	0.880
Infiltration	0.636	0.673	0.700	0.700	0.709	0.677	0.696	0.711	**0.715**	0.713
Mass	0.761	0.718	0.810	0.830	0.821	0.783	0.838	**0.857**	0.855	0.847
Nodule	0.664	0.777	0.759	0.750	0.758	0.698	0.771	0.791	**0.798**	0.788
Pneumonia	0.664	0.684	0.718	0.670	0.731	0.696	0.722	0.775	**0.777**	0.775
Pneumothorax	0.799	0.805	0.848	0.870	0.846	0.810	0.862	0.875	**0.886**	0.884
Consolidation	0.770	0.711	0.741	0.800	0.745	0.726	0.750	0.814	**0.817**	0.815
Edema	0.861	0.806	0.844	0.880	0.835	0.833	0.846	0.900	**0.902**	0.897
Emphysema	0.736	0.842	0.891	0.910	0.895	0.822	0.908	0.929	**0.934**	0.932
Fibrosis	0.739	0.743	0.810	0.780	0.818	0.804	0.827	0.848	**0.856**	0.841
PT	0.749	0.724	0.768	0.790	0.761	0.751	0.779	0.790	**0.791**	0.789
Hernia	0.746	0.775	0.867	0.770	0.896	0.900	0.934	**0.953**	0.947	0.936
Average	0.758	0.761	0.801	0.806	0.807	0.781	0.817	0.847	**0.850**	0.845

**Note:**

The best results are marked in bold.

As shown in Table 1, the multi-scale attention model has achieved the best results in terms of the average AUROC scores for most individual diseases. The proposed model induced significant improvement, especially for an unbalanced disease class where positive samples are rare. For instance, in the dataset, 'Hernia' has only 227 pictures (0.202 %), and ‘Emphysema’ has only 2,516 pictures (2.244 %). The proposed MS-ANet_2_ model obtains outstanding AUROC values, 0.947 and 0.934, respectively, for Hernia and Emphysema, outperforming other methods. It is mainly due to the employed multi-scale attention loss, punishing those samples that are difficult to identify so that negative and positive samples can be distinguished better.

#### Ablation experiment

In order to evaluate the contribution of each module of the proposed model, ablation experiments were conducted by comparing the impact on classification accuracy. In Table 2, Densenet121 and Densenet169 are denoted as ‘121’ and ‘169’, respectively. The binary cross-entropy, multi-label balance, and perceptual losses are denoted as L_bce_, L_bal_, and L_per_, respectively.

**Table 2:** Results comparison between different methods.

Methods		Methods	
121 + $L_{bce}$	0.801	169 (MS-FIF) + $L_{bal}$	0.839
121 + $L_{bal}$	0.806	121 (MS-FIF) + $L_{bal}$ + $L_{per}$	0.847
121 (MS-FIF) + $L_{bal}$	0.840	121+169 (MS-FIF) + $L_{bal}$ + $L_{per}$	0.850
121+169 (MS-FIF) + $L_{bal}$	0.842	169 (MS-FIF) + $L_{bal}$ + $L_{per}$	0.845

Table 2 shows that the proposed MS-ANet improves the accuracy in the unbalanced dataset. The AUROC score is increased by 0.032 on average with the MS-FIF, obtaining 0.840 from 0.806. Since through a more collaborative way through each other's s reverse propagation gradient to update. The AUROC score is increased to 0.847 with adopting the multi-scale perceptual loss. Since the perceptual loss narrows the attention of the two networks, thus promoting the cross-attention. The proposed MS-ANet obtains 0.847, 0.850, and 0.845 AUROC, which is the latest and most advanced result.

#### Image-level supervised disease localization

Based on the quantitative analysis of the classification performance of the model, this paper makes a qualitative analysis of the model. The localization heat map of the lesion area is generated by the CAM, which is used to visually explain the lesion area on which the network is based when judging the disease. The product of weight and feature mapping of the fully-connected layer and pooling layer is directly located because of the global average pooling used as the final pooling layer.

**Figure 4:**
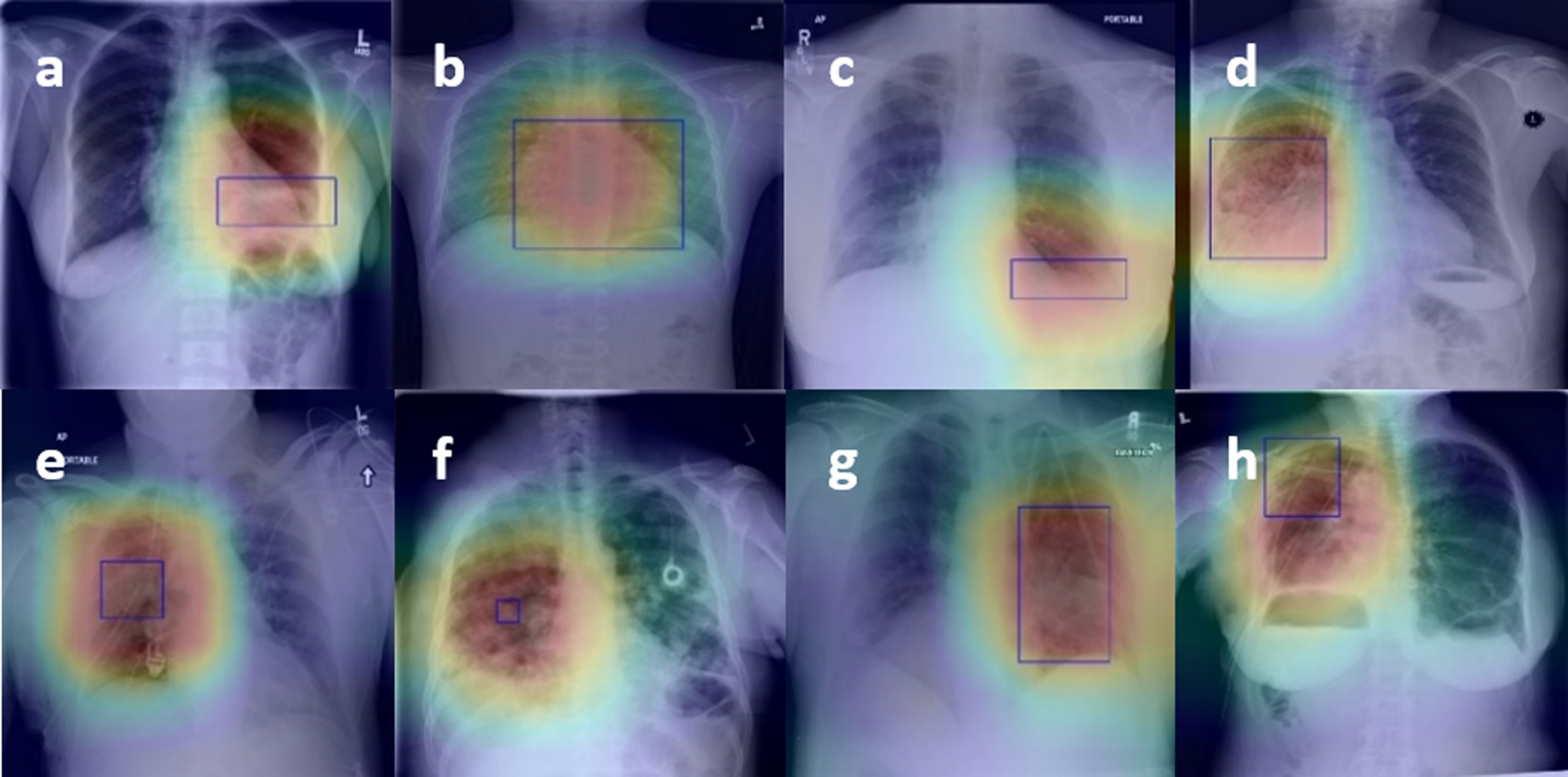
Figure 4. The results of the localization of chest diseases. (A) Atelectasis, (B) Cardiomegaly, (C) Effusion, (D) Infiltration, (E) Mass, (F) Nodule, (G) Pneumonia, (H) Pneumothorax.

Figure 4 presents the localization results of eight diseases, in which the bright red part is the main area for diagnosis, and the blue boundary frame indicates the reallocation of the disease. After observation, whether it is a large disease in the lesion area (such as Fig. 4B Cardiomegaly) or a small disease in the lesion area (such as Fig. 4F Nodule), the lesion area located by the thermal map can be well consistent with the lesion area annotation box given by the doctor, which further verifies that the feature information based on the network in the diagnosis is accurate and effective. Besides, visualization of lesion areas can provide better visual support for professional doctors by computer-aided diagnosis in clinical application, obtain doctors' trust, and help doctors to make a rapid and accurate diagnosis.

### Results on CheXpert Dataset

#### Dataset and training

The CheXpert (Irvin et al., 2019) dataset, which contains large-scale data of 224,316 chest radiographs of 65,240 patients with frontal and lateral radiographs, is also used to validate the proposed model. The experiment was conducted as similar to the experiment on the Chest X-Ray14 dataset in all comparative experiments. The images are divided into 222,914 images for the training set and 734 images for the testing set. No duplication is ensured between those images.

#### Comparison analysis

In this experiment, the U(uncertain)-One introduced in CheXpert’ s paper (Irvin et al., 2019) is only used to maintain consistent experimental settings, labeling all uncertain samples to 1. Besides, we tested the models for fourteen labels and divided them into using only the frontal radiographs, and using the frontal and lateral radiographs equally. The classification results for fourteen classes are compared and summarized in Table 3. As shown in Table 3, the proposed model obtains the highest AUROC scores (marked in bold) for most classes, including both the frontal radiographs and the frontal/lateral radiographs.

**Table 3:** Results comparison on 14 labels classification tasks on CheXpert dataset.

Experiments	Frontal views only			Frontal + Lateral (Equally)		
Labels	CheXNet	Ho & Gwak (2019)	MS-ANet	CheXNet	Ho & Gwak (2019)	MS-ANet
Atelectasis	0.659	0.667	**0.793**	0.707	0.713	**0.793**
Cardiomegaly	0.775	0.773	**0.818**	0.775	**0.790**	0.818
Consolidation	0.702	0.732	**0.923**	0.755	0.757	**0.923**
Edema	0.827	0.840	**0.928**	0.863	0.861	**0.928**
Enlarged Cardio	0.551	**0.552**	0.541	0.531	**0.555**	0.541
Fracture	0.616	0.722	**0.918**	0.588	0.735	**0.918**
Lung Lesion	0.704	0.757	0.288	0.710	**0.805**	0.288
Lung Opacity	0.767	0.788	**0.921**	0.784	0.783	**0.921**
No Finding	0.887	**0.893**	0.864	0.872	0.859	**0.889**
Pleural Effusion	0.860	0.892	0.919	0.874	0.892	**0.919**
Pleural Other	0.607	0.711	**0.979**	0.710	0.680	**0.979**
Pneumonia	0.641	**0.710**	0.645	0.535	**0.666**	0.645
Pneumothorax	0.807	0.824	**0.889**	0.842	0.836	**0.889**
Support Devices	0.869	0.889	**0.956**	0.899	0.913	**0.956**
Average	0.734	0.768	**0.813**	0.746	0.775	**0.815**

**Note:**

The highest AUROC scores are marked in bold.

## Conclusion

In this paper, An end-to-end learning framework, Multi-Scale Attention Network (MS-ANet), is proposed to address multi-class X-ray chest disease recognition. MS-ANet not only makes full use of image information at different scales to obtain better feature regions through iterative fusion but also addresses the negative/positive sample imbalance problem and controlled simple class samples. MS-ANet updates the model through iterative fusion and more collaborative ways, effectively extracting significant feature expressions. Quantitative and qualitative results show that our method achieves the state-of-the-art effect, 0.850 and 0.815 AUROC respectively. This study has important practical significance for the use of AI algorithm in assisting radiologists to improve work efficiency and diagnostic accuracy, which is helpful to reduce the pressure of doctors in metropolitan hospitals and improve the diagnostic quality in rural areas.

## Supplemental Information

10.7717/peerj-cs.541/supp-1 Supplemental Information 1 Code by Python.

## Additional Information and Declarations

### Data Availability

The following information was supplied regarding data availability: CheXpert: CheXpert Contains large-scale data of 224,316 chest radiographs of 65,240 patients (including “front view” and “side view” X-ray images,14 disease). https://stanfordmlgroup.github.io/competitions/chexpert/. Python scripts are available in the Supplemental File. Chest X-Ray14: Chest X-ray dataset comprises 112,120 frontal-view X-ray images of 30,805 unique patients with the text-mined fourteen disease image labels (where each image can have multi-labels), mined from the associated radiological reports using natural language processing. https://nihcc.app.box.com/v/ChestXray-NIHCC.
